# Associations of Homocysteine, Folate, and Vitamin B12 with Osteoarthritis: A Mendelian Randomization Study

**DOI:** 10.3390/nu15071636

**Published:** 2023-03-28

**Authors:** Haofeng Hong, Longting Chen, Yiming Zhong, Zihuan Yang, Weishi Li, Chunli Song, Huijie Leng

**Affiliations:** 1 Department of Orthopaedics, Peking University Third Hospital, Beijing 100191, China; 2 Engineering Research Center of Bone and Joint Precision Medicine, Ministry of Education Lisbon Portugal, Beijing 100191, China; 3 Beijing Key Lab of Spine Diseases, Beijing 100191, China

**Keywords:** homocysteine, folate, vitamin B12, osteoarthritis, Mendelian randomization

## Abstract

Homocysteine, inversely related to folate and vitamin B12, is an independent risk factor for several age-related disorders. However, little is known about the association of homocysteine and related vitamins with osteoarthritis (OA). This study aimed to elucidate the potential causal effects of homocysteine, folate, and vitamin B12 on site- and gender-specific OA by applying the two-sample Mendelian randomization (MR) approach. Genetically predicted homocysteine showed adverse effects on overall OA (95% confidence interval (CI): 1.044–1.155), knee OA (95% CI: 1.000–1.167), hip OA (95% CI: 1.057–1.297), and spine OA (95% CI: 1.017–1.216). Genetically predicted folate showed protective effects on overall OA (95% CI: 0.783–0.961) and spine OA (95% CI: 0.609–0.954). Folate (95% CI: 0.887–1.004) and vitamin B12 (95% CI: 0.886–1.009) showed a protective trend against knee OA. The patterns of associations were site and gender specific. In conclusion, homocysteine had adverse effects on OA, especially on OA at weight-bearing joints and in females. Folate and vitamin B12 had protective effects on OA. Homocysteine-lowering interventions may be a potential option in the treatment and prevention of OA.

## 1. Introduction

Osteoarthritis (OA) is a common joint disease characterized by cartilage damage and degradation of other tissues, such as subchondral bone within the joint [1]. OA is an age- and sex-related disorder with stiffness, pain, and impaired joint movement as the main symptoms. Studies have shown that the risk of OA increases rapidly between the ages of 50 years and 75 years and that it is more prevalent in women than in men [1,2]. OA can occur at all joints in the body. Notably, different patterns of OA have been demonstrated in different sites and genders in humans [2]. Although some symptomatic treatments have been recommended to reduce pain and improve joint movement, effective conservative treatments to halt or cure cartilage damage in OA are still currently unavailable, partly due to the limited understanding of the pathogenesis of OA [3].

Homocysteine, an intermediate metabolite within the methionine cycle, can be elevated in blood by deficiency of the related B vitamins, mainly folate and vitamin B12 [4]. A meta-analysis in China with 60,754 subjects aged 3–97 years demonstrated that the prevalence of hyperhomocysteinemia (HHcy, total homocysteine concentrations more than 15 μmol/L in blood) increased with age and was the highest in the group of people older than 65 years [4]. Higher blood concentrations of homocysteine have been identified as a risk factor for several age-related disorders, including cardiovascular disease (CVD) and osteoporosis [5,6]. Recently, in vitro studies revealed that homocysteine can stimulate osteoclast differentiation and cause chondrocyte dysfunction [7,8]. Considering the changes with aging for both OA and homocysteine and the potential relation between homocysteine and joint metabolism, a possible causal association of homocysteine with OA could be inferred. However, to date, no randomized controlled trials (RCTs) have been reported on the causal relationship between blood homocysteine and OA. Moreover, only a few observational studies have focused on the correlation between blood homocysteine levels and OA in humans, and the limited data provided inconsistent conclusions [9,10,11,12]. The Framingham OA study showed that homocysteine levels were not associated with the radiological incidence and progression of knee OA in women [10]. A cross-sectional study in Japan observed that homocysteine levels were related to the prevalence rate of spinal OA in postmenopausal women [9]. Another study in China observed a significantly elevated serum homocysteine level in patients with severe OA [12]. These inconsistent results are probably due to residue bias, confounding factors, and reverse causality. Thus, the exact causal relationship between blood homocysteine and OA is still unclear and deserves further investigation.

The Mendelian randomization (MR) approach is able to circumvent the disadvantages of the observational studies mentioned above by applying genetic variants as instrumental variables (IVs) and has been widely applied to evaluate the causal association of exposures with outcomes [13,14]. The present study aimed to examine the causal association of homocysteine and B vitamins with site- and gender-specific OA using the MR approach.

## 2. Materials and Methods

### 2.1. Study Design

**Figure 1:**
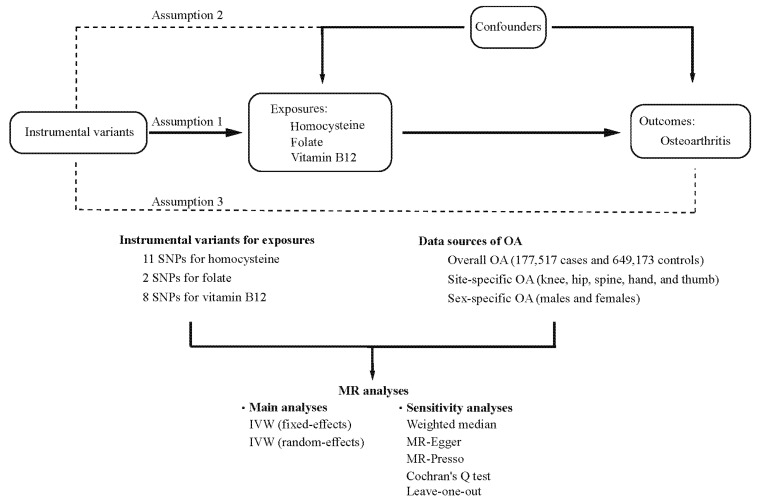
An overview of the study design. SNP: single nucleotide polymorphism; OA: osteoarthritis; MR: Mendelian randomization; IVW: inverse-variance weighted.

MR analyses have 3 key assumptions: (1) IVs are strongly related to the exposure; (2) IVs should not be related to potential confounders; and (3) IVs should affect the outcome exclusively through the exposure. A sketch of the study design is illustrated in Figure 1.

### 2.2. Data Sources of Outcomes

Genetic data for OA were derived from the largest GWAS (genome-wide association study) to date by Boer’s group [15], including overall OA (177,517 cases and 649,173 controls) and 5 different sites of OA (knee OA, hip OA, spine OA, hand OA, and thumb OA) (Table S1). In addition, sex-specific analysis was conducted in this GWAS meta-analysis. Given that sex is related to both OA and homocysteine, genetic data for OA in each sex were also extracted from these GWAS.

### 2.3. Selection of Instrumental Variables

SNPs related to homocysteine, folate, and vitamin B12 were extracted from two previous GWASs, which included 44,147, 37,465, and 45,576 individuals of European ancestry [16,17].

IVs were selected according to the following criteria: (1) independent SNPs (r^2^ = 0.1, KB = 10,000) with genome-wide significance (*p* < 5 × 10^−8^) [18]; (2) nonrare SNPs (MAF ≥ 0.01); (3) SNPs unrelated to potential confounders by checking each of the SNPs in the PhenoScanner database (http://www.phenoscanner.medschl.cam.ac.uk/, accessed on 4 December 2022, Table S2) [19]; and (4) SNPs without reverse causality by performing the MR Steiger filtering test (Table S3) [20]. Finally, 11 SNPs for homocysteine, 2 SNPs for folate, and 8 SNPs for vitamin B12 were selected in MR analyses. Summary statistics of the selected SNPs are shown in Table 1 and Table S4.

### 2.4. MR Analyses

All analyses were performed in R software (version 4.1.3) using the R package “TwoSampleMR” [21]. The inverse-variance weighted (IVW) method based on the fixed-effects and random-effects model was utilized as the main analysis [22,23]. The weighted median and MR-Egger methods, which make diverse assumptions about horizontal pleiotropy, were performed as complementary methods to test the robustness of the main analysis [24,25]. Cochrane’s Q value was used to assess the heterogeneity among estimates of SNPs [26]. The intercept in MR-Egger regression and MR pleiotropy residual sum and outlier (MR-PRESSO) test were applied to identify pleiotropy [27]. The leave-one-out method was implemented by sequentially excluding each SNP to determine whether the estimates were driven by any single SNP.

## 3. Results

### 3.1. Causal Effects of Homocysteine, Folate, and Vitamin B12 on OA

**Figure 2:**
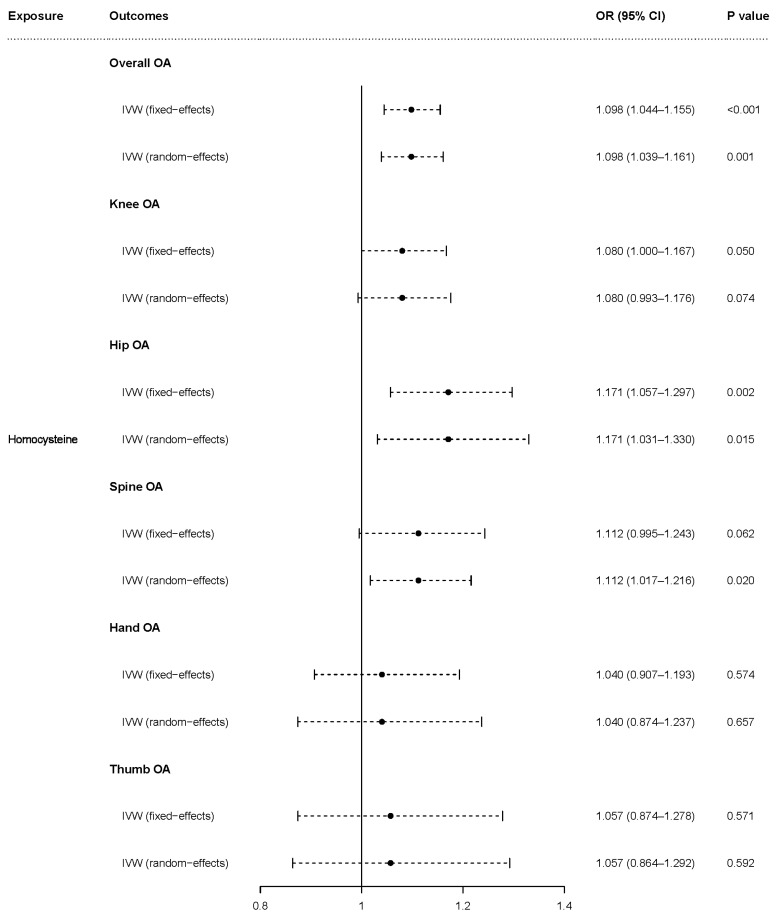
Causal effect of homocysteine on OA and subtypes in fixed-effects and random-effects IVW analyses. OR: odds ratio; CI: confidence interval; *p* value: *p* value of the causal estimate.

The fixed-effects model of IVW analyses showed that genetically predicted homocysteine was causally associated with overall OA [odds ratio (OR) = 1.098, 95% (CI) = 1.044–1.155, *p* < 0.001], knee OA (OR = 1.080, 95% CI = 1.000–1.167, *p* = 0.050), and hip OA (OR = 1.171, 95% CI = 1.057–1.297, *p* = 0.002) but was not associated with hand OA (OR = 1.040, 95% CI = 0.907–1.193, *p* = 0.574) or thumb OA (OR = 1.057, 95% CI = 0.874–1.278, *p* = 0.571). The causal association of homocysteine with spine OA closely approached statistical significance (OR = 1.112, 95% CI = 0.995–1.243, *p* = 0.062) based on the fixed-effects model. The random-effects model, which demonstrated similar results to the fixed-effects model, showed that genetically predicted homocysteine was causally associated with overall OA (OR = 1.098, 95% CI = 1.039–1.161, *p* = 0.001), hip OA (OR = 1.171, 95% CI = 1.031–1.330, *p* = 0.015), and spine OA (OR = 1.112, 95% CI = 1.017–1.216, *p* = 0.020) but was not associated with hand OA (OR = 1.040, 95% CI = 0.874–1.237, *p* = 0.657) or thumb OA (OR = 1.057, 95% CI = 0.864–1.292, *p* = 0.592). Higher homocysteine levels tended to increase the risk of knee OA (OR = 1.080, 95% CI = 0.993–1.176, *p* = 0.074) based on the random-effects model (Figure 2).

**Figure 3:**
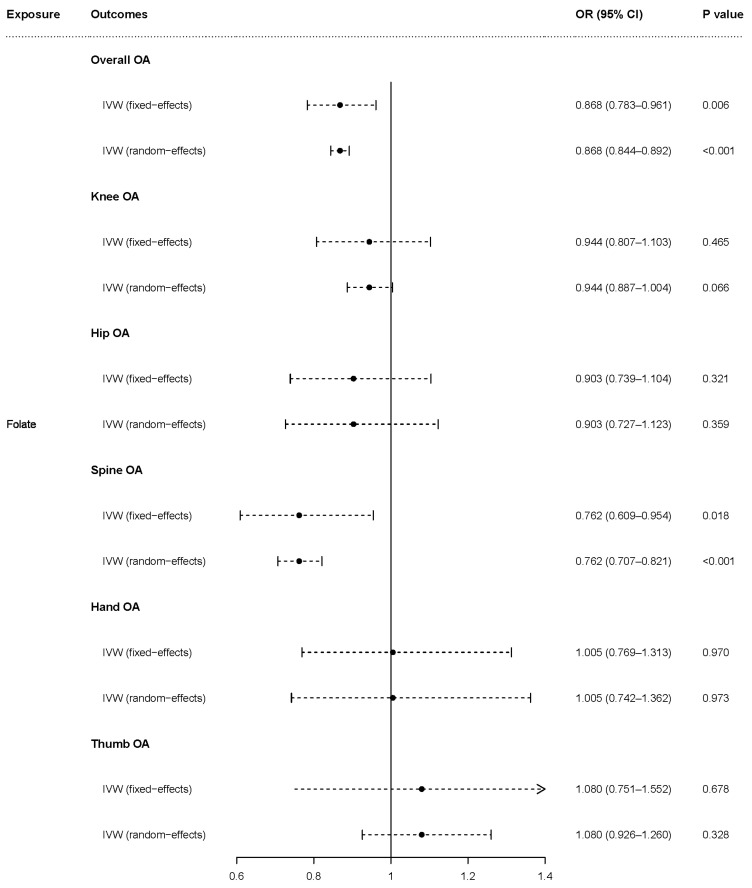
Causal effect of folate on OA and subtypes in fixed-effects and random-effects IVW analyses. OR: odds ratio; CI: confidence interval; *p* value: *p* value of the causal estimate.

The fixed-effects model of IVW analyses showed that genetically predicted folate was causally associated with overall OA (OR = 0.868, 95% CI = 0.783–0.961, *p* = 0.006) and spine OA (OR = 0.762, 95% CI = 0.609–0.954, *p* = 0.018) but was not associated with knee OA (OR = 0.944, 95% CI = 0.807–1.103, *p* = 0.465), hip OA (OR = 0.903, 95% CI = 0.739–1.104, *p* = 0.321), hand OA (OR = 1.005, 95% CI = 0.769–1.313, *p* = 0.970), or thumb OA (OR = 1.080, 95% CI = 0.751–1.552, *p* = 0.678). The random-effects model showed that genetically predicted folate was causally associated with overall OA (OR = 0.868, 95% CI = 0.844–0.892, *p* < 0.001) and spine OA (OR = 0.762, 95% CI = 0.707–0.821, *p* < 0.001) but not was associated with hip OA (OR = 0.903, 95% CI = 0.727–1.123, *p* = 0.359), hand OA (OR = 1.005, 95% CI = 0.742–1.362, *p* = 0.973), or thumb OA (OR = 1.080, 95% CI = 0.926–1.260, *p* = 0.328). The causal association of folate with knee OA closely approached statistical significance (OR = 0.944, 95% CI = 0.887–1.004, *p* = 0.066) based on the random-effects model (Figure 3).

**Figure 4:**
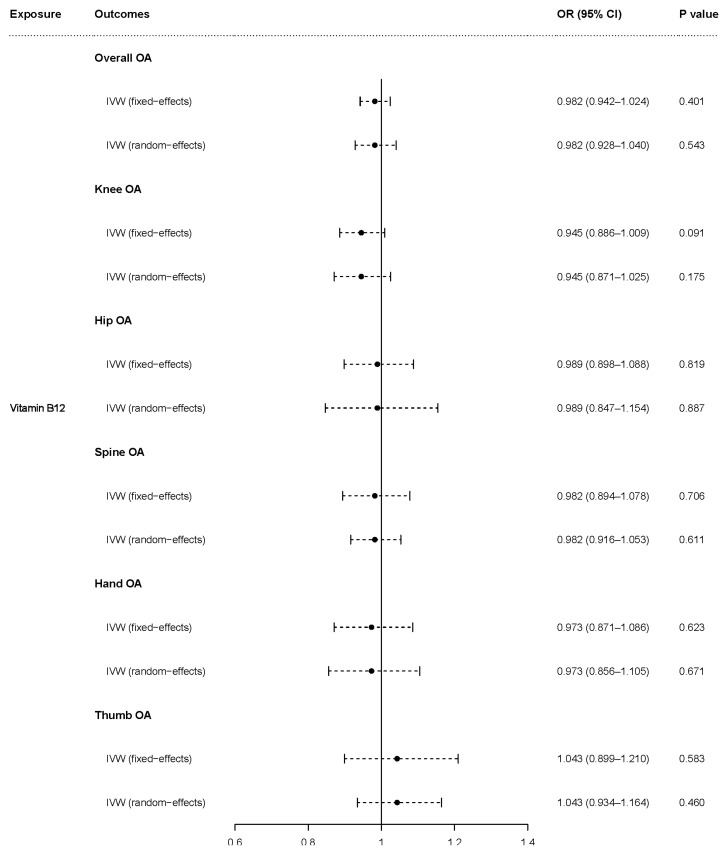
Causal effect of vitamin B12 on OA and subtypes in fixed-effects and random-effects IVW analyses. OR: odds ratio; CI: confidence interval; *p* value: *p* value of the causal estimate.

The fixed-effects model of IVW analyses showed that genetically predicted vitamin B12 tended to be protective against knee OA (OR = 0.945, 95% CI = 0.886–1.009, *p* = 0.091) and genetically predicted vitamin B12 was not associated with overall OA (OR = 0.982, 95% CI = 0.942–1.024, *p* = 0.401), hip OA (OR = 0.989, 95% CI = 0.898–1.088, *p* = 0.819), spine OA (OR = 0.982, 95% CI = 0.894–1.078, *p* = 0.706), hand OA (OR = 0.973, 95% CI = 0.871–1.086, *p* = 0.623), or thumb OA (OR = 1.043, 95% CI = 0.899–1.210, *p* = 0.583). The random-effects model demonstrated that there was no association of genetically predicted vitamin B12 with overall OA (OR = 0.982, 95% CI = 0.928–1.040, *p* = 0.543), knee OA (OR = 0.945, 95% CI = 0.871–1.025, *p* = 0.175), hip OA (OR = 0.989, 95% CI = 0.847–1.154, *p* = 0.887), spine OA (OR = 0.982, 95% CI = 0.916–1.053, *p* = 0.611), hand OA (OR = 0.973, 95% CI = 0.856–1.105, *p* = 0.671), or thumb OA (OR = 1.043, 95% CI = 0.934–1.164, *p* = 0.460) (Figure 4).

Cochran’s Q (P_Cochran’s Q_ > 0.05), the MR-Egger intercept test (P_intercept_ > 0.05), and the MR-PRESSO global test (P_global test_ > 0.05) demonstrated no heterogeneity and no substantial horizontal pleiotropy, except for the association of vitamin B12 with hip OA (Tables S5 and S6). The results of the weighted median method, scatter plots, and leave-one-out analysis are shown in Table S7 and Figures S1–S4.

### 3.2. Causal Effects of Homocysteine, Folate, and Vitamin B12 on OA in Females

In females, the fixed-effects model of IVW analyses showed that genetically predicted homocysteine was associated with overall OA (OR = 1.090, 95% CI = 1.016–1.169, *p* = 0.016) and knee OA (OR = 1.137, 95% CI = 1.022–1.264, *p* = 0.018) but was not associated with hand OA (OR = 0.982, 95% CI = 0.807–1.193, *p* = 0.852) or thumb OA (OR = 1.003, 95% CI = 0.736–1.367, *p* = 0.983). The causal association of homocysteine with hip OA (OR = 1.137, 95% CI = 0.992–1.303, *p* = 0.064), as well as spine OA (OR = 1.156, 95% CI = 0.997–1.341, *p* = 0.055), closely approached statistical significance based on the fixed-effects model. The random-effects model showed that genetically predicted homocysteine was associated with overall OA (OR = 1.090, 95% CI = 1.039–1.143, *p* < 0.001), knee OA (OR = 1.137, 95% CI = 1.024–1.262, *p* = 0.016), hip OA (OR = 1.137, 95% CI = 1.027–1.258, *p* = 0.013), and spine OA (OR = 1.156, 95% CI = 1.048–1.275, *p* = 0.004) but was not associated with hand OA (OR = 0.982, 95% CI = 0.761–1.266, *p* = 0.886) or thumb OA (OR = 1.003, 95% CI = 0.804–1.252, *p* = 0.977) (Figure S5).

The fixed-effects model of IVW analyses demonstrated that there was no evidence for associations of folate with overall OA (OR = 0.902, 95% CI = 0.782–1.041, *p* = 0.159), knee OA (OR = 0.933, 95% CI = 0.752–1.159, *p* = 0.532), hip OA (OR = 0.953, 95% CI = 0.722–1.258, *p* = 0.734), spine OA (OR = 0.783, 95% CI = 0.580–1.059, *p* = 0.112), hand OA (OR = 0.961, 95% CI = 0.646–1.429, *p* = 0.844), and thumb OA (OR = 1.259, 95% CI = 0.671–2.361, *p* = 0.474) in females. The random-effects model showed that genetically predicted folate was inversely associated with overall OA (OR = 0.902, 95% CI = 0.821–0.991, *p* = 0.032) and knee OA (OR = 0.933, 95% CI = 0.929–0.938, *p* < 0.001), and the causal association of folate with spine OA (OR = 0.783, 95% CI = 0.598–1.026, *p* = 0.076) closely approached statistical significance. There was no evidence for associations of folate with hip OA (OR = 0.953, 95% CI = 0.711–1.276, *p* = 0.746), hand OA (OR = 0.961, 95% CI = 0.706–1.309, *p* = 0.801), and thumb OA (OR = 1.259, 95% CI = 0.826–1.917, *p* = 0.284) based on the random-effects model (Figure S6). 

The fixed-effects model of IVW analyses showed that the causal association of vitamin B12 with knee OA in females (OR = 0.901, 95% CI = 0.808–1.004, *p* = 0.060) closely approached statistical significance, and there was no association of genetically predicted vitamin B12 with overall OA (OR = 0.972, 95% CI = 0.906–1.044, *p* = 0.437), hip OA (OR = 0.998, 95% CI = 0.868–1.147, *p* = 0.974), spine OA (OR = 1.012, 95% CI = 0.869–1.178, *p* = 0.880), hand OA (OR = 0.878, 95% CI = 0.719–1.074, *p* = 0.206), and thumb OA (OR = 0.973, 95% CI = 0.708–1.339, *p* = 0.868). The random-effects model showed that genetically predicted vitamin B12 was associated with knee OA (OR = 0.901, 95% CI = 0.822–0.987, *p* = 0.025) but was not associated with overall OA (OR = 0.972, 95% CI = 0.919–1.028, *p* = 0.325), hip OA (OR = 0.998, 95% CI = 0.864–1.152, *p* = 0.974), spine OA (OR = 1.012, 95% CI = 0.876–1.169, *p* = 0.873), hand OA (OR = 0.878, 95% CI = 0.721–1.070, *p* = 0.198), or thumb OA (OR = 0.973, 95% CI = 0.742–1.277, *p* = 0.846) (Figure S7).

### 3.3. Causal Effects of Homocysteine, Folate, and Vitamin B12 on OA in Males

In males, the fixed-effects model of IVW analyses showed that the causal association of homocysteine was related to overall OA (OR = 1.132, 95% CI = 1.042–1.229, *p* = 0.003), and the causal association of homocysteine with hip OA (OR = 1.171, 95% CI = 0.993–1.380, *p* = 0.061) closely approached statistical significance. There was no evidence for associations of homocysteine with knee OA (OR = 1.042, 95% CI = 0.921–1.180, *p* = 0.512), spine OA (OR = 1.098, 95% CI = 0.919–1.312, *p* = 0.305), hand OA (OR = 0.978, 95% CI = 0.716–1.336, *p* = 0.888), and thumb OA (OR = 1.073, 95% CI = 0.634–1.816, *p* = 0.792) based on the fixed-effects model. The random-effects model showed that genetically predicted homocysteine was associated with overall OA (OR = 1.132, 95% CI = 1.040–1.232, *p* = 0.004) but was not associated with knee OA (OR = 1.042, 95% CI = 0.914–1.189, *p* = 0.536), hip OA (OR = 1.171, 95% CI = 0.938–1.460, *p* = 0.163), spine OA (OR = 1.098, 95% CI = 0.907–1.329, *p* = 0.338), hand OA (OR = 0.978, 95% CI = 0.694–1.377, *p* = 0.898), or thumb OA (OR = 1.073, 95% CI = 0.538–2.143, *p* = 0.841) (Figure S8).

The fixed-effects model of IVW analyses showed that the causal association of folate was related to overall OA (OR = 0.792, 95% CI = 0.668–0.939, *p* = 0.007), and the causal association of folate with spine OA (OR = 0.737, 95% CI = 0.512–1.061, *p* = 0.100) closely approached statistical significance in males. No association was observed between folate and knee OA (OR = 0.886, 95% CI = 0.687–1.143, *p* = 0.351), hip OA (OR = 0.828, 95% CI = 0.591–1.160, *p* = 0.272), hand OA (OR = 1.221, 95% CI = 0.644–2.316, *p* = 0.541), or thumb OA (OR = 0.537, 95% CI = 0.180–1.606, *p* = 0.266) based on the fixed-effects model. The random-effects model demonstrated that genetically predicted folate was inversely associated with overall OA (OR = 0.792, 95% CI = 0.769–0.815, *p* < 0.001), knee OA (OR = 0.886, 95% CI = 0.788–0.996, *p* = 0.042), and spine OA (OR = 0.737, 95% CI = 0.569–0.954, *p* = 0.021), and the causal association of folate with hip OA (OR = 0.828, 95% CI = 0.661–1.037, *p* = 0.100) closely approached statistical significance. No association was observed between folate and hand OA (OR = 1.221, 95% CI = 0.881–1.692, *p* = 0.230) or thumb OA (OR = 0.537, 95% CI = 0.115–2.514, *p* = 0.430) based on the random-effects model (Figure S9).

The fixed-effects model of IVW analyses showed that the causal association of vitamin B12 with knee OA in males (OR = 0.891, 95% CI = 0.785–1.011, *p* = 0.073) closely approached statistical significance, and genetically predicted vitamin B12 was not associated with overall OA (OR = 0.971, 95% CI = 0.894–1.055, *p* = 0.489), hip OA (OR = 1.058, 95% CI = 0.894–1.251, *p* = 0.511), spine OA (OR = 0.922, 95% CI = 0.770–1.104, *p* = 0.378), hand OA (OR = 0.960, 95% CI = 0.699–1.318, *p* = 0.799), or thumb OA (OR = 0.918, 95% CI = 0.540–1.561, *p* = 0.752). The random-effects model showed that vitamin B12 was associated with knee OA (OR = 0.891, 95% CI = 0.818–0.971, *p* = 0.008) but not with overall OA (OR = 0.971, 95% CI = 0.879–1.073, *p* = 0.566), hip OA (OR = 1.058, 95% CI = 0.768–1.458, *p* = 0.731), spine OA (OR = 0.922, 95% CI = 0.818–1.040, *p* = 0.187), hand OA (OR = 0.960, 95% CI = 0.675–1.364, *p* = 0.818), or thumb OA (OR = 0.918, 95% CI = 0.663–1.270, *p* = 0.605) (Figure S10). 

## 4. Discussion

In this MR study, we investigated the causal effects of homocysteine, folate, and vitamin B12 on the risk of overall and site- and gender-specific OA. The results revealed suggestive associations of higher genetically predicted homocysteine levels with increased risk of overall OA and OA at weight-bearing joints (knee, hip, and spine), especially in females. Folate and vitamin B12 had protective effects on knee OA in both males and females. Furthermore, folate was also associated with a lower risk of overall OA and spine OA in both males and females.

The present study shows that higher homocysteine levels could increase the risk of overall OA. The reason for this result is still unclear due to the lack of studies investigating the pathogenic mechanisms of homocysteine in OA. Based on the reported adverse effects of homocysteine in the literature, a possible association of homocysteine with OA could be inferred. A recent in vitro study reported that homocysteine could induce several detrimental changes in chondrocytes including mitochondrial dysfunction, apoptosis, and oxidative stress accumulation [8]. Moreover, accumulated evidence has suggested that homocysteine could affect bone metabolism and reduce bone strength [7]. Homocysteine in bone was not only bound to collagen of the extracellular matrix but also disturbed the osteoblast-mediated calcification process and stimulated osteoclast differentiation. In diet-induced HHcy in rats, homocysteine accumulated in bone was accompanied by bone loss and bone strength reduction [28]. It is well known that subchondral bone and cartilage form a functional complex called the bone–cartilage unit, which is involved in the pathophysiology of OA biochemically and mechanically [1]. Subchondral bone is an important aspect of the mechanical environment for cartilage, and its microstructure has close relationship with cartilage metabolism [29,30]. Although there are different microstructural changes in subchondral bone at different stages of OA (enhanced subchondral bone turnover in early OA and subchondral bone sclerosis in the advanced stage), subchondral bone in OA undergoes an uncoupling process at different stages during OA progression [1]. The equilibrium between osteoclast-mediated bone resorption and osteoblast-mediated bone formation is crucial for bone homeostasis, and spatiotemporal uncoupling is responsible for the degradation of subchondral bone and progression of OA [30]. These findings suggest that homocysteine plays adverse roles in collagen metabolism, chondrocyte function, and bone formation and may be involved in OA pathophysiology.

Notably, the causal effects of homocysteine on OA were site specific. Homocysteine was associated with OA in weight-bearing joints (knee, hip and spine) but not with hand OA and thumb OA. OA itself is site specific with heterogeneity in terms of prevalence, epidemiological risk factors, genetics, and etiology [1,31,32]. Mechanical factors causing the wear of cartilage and affecting the integrity of subchondral bone have been considered one of the most important factors ascribed to the site specificity of OA [33]. There are three main patterns of hand OA: the symmetrical pattern (same joint involvement from left and right hands), the row pattern (several distal interphalangeal (DIP) or proximal interphalangeal (PIP) joint involvement), and the ray pattern (DIP/PIP joint involvement of the same finger). The progression of hand OA demonstrated in a symmetrical pattern, which mainly resulted from systemic factors rather than mechanical factors [33]. However, unlike the hand joint, which is thought to be influenced mostly by systemic factors, weight-bearing joints (knee, hip, and spine) present a distinct biomechanical environment and are more susceptible to mechanical changes [34]. Mechanical factors may explain our MR results regarding homocysteine’s prominent effects in weight-bearing joints. Among the periarticular tissues, subchondral bone contributes significantly to the mechanical environment and plays different roles in the onset and progression of OA at different sites. Thus, the differential effects of homocysteine on OA in weight-bearing and non-weight-bearing joints suggest that blood homocysteine may be involved in OA pathogenesis not only through directly disturbing chondrocyte functions but also through affecting subchondral bone.

The sex-stratified MR analyses demonstrated that the causal effects of homocysteine on OA were gender specific. For females, homocysteine was associated with overall OA, knee OA, hip OA, and spine OA but not with hand OA and thumb OA. For males, homocysteine was only associated with overall OA and hip OA. Previous studies also reported sex specificity regarding the association between homocysteine and OA [9,10]. Both HHcy and OA are well-known sex-related disorders. Epidemiological studies have reported an increased prevalence of OA and HHcy in postmenopausal women. Estrogen deficiency could increase blood levels of homocysteine and induce or worsen OA [35,36,37]. Among the periarticular tissues, the subchondral bone responds most significantly to estrogen deficiency, exhibiting increased bone turnover accompanied by reduced stiffness and altered mechanical characteristics [37]. The sex-specific pattern of association seems to provide another clue that blood homocysteine levels influence OA development through bone structural alteration. Taking the site- and sex-specific patterns of homocysteine’s effects on OA together, subchondral bone is nonnegligible during OA development stimulated by homocysteine. The exact mechanisms need to be further investigated.

Since folate and vitamin B12 supplementation are well-recognized methods to reduce the risk of diseases by lowering the blood level of homocysteine, the causal effects of folate and vitamin B12 on OA were evaluated in the present study. The protective and site-specific effects of folate and vitamin B12 on OA were manifested. Sex-stratified analyses showed similar patterns in males and females. In line with our findings, previous studies also demonstrated an inverse effect of folate and vitamin B12 on OA. Folate deficiency was reported to be associated with increased radiographic severity of knee OA [38]. Knee OA patients had lower intakes of dietary folate than controls [39]. Dietary folate and vitamin B12 supplementation can improve joint functions in OA patients [40]. Rationally, folate and vitamin B12 supplementation may be an optional intervention to prevent OA.

People remain unconvinced about whether and how homocysteine and human OA are related. To date, no related RCTs have been conducted. Traditional observational and cohort studies have demonstrated inconsistent results, probably due to some confounding factors. The Framingham osteoarthritis study concluded that homocysteine was not associated with the progression of radiographic knee OA [10], whereas cross-sectional studies in Japan and China observed the correlation of circulating homocysteine levels with spinal OA and knee OA, respectively [9,12]. A recent MR study indicated that hip OA is not correlated with higher homocysteine concentrations and the related B vitamins have no causal effects on OA [41]. These results seem different from ours, which might be attributed to the choice of outcome data sources and selection of SNPs. Our MR study used the largest GWAS for OA to date, which contains the data of site- and gender-specific OA, and selected instrumental variables complying with three key assumptions in MR design. The present design might provide more comprehensive results about the potential association of homocysteine and the related B vitamins with OA.

There are several limitations in the present study. First, sensitivity analyses of the association of folate with outcomes could not be performed since only two SNPs after screening were available for folate. Second, since the individuals included in our study were mainly European individuals, our findings may not be directly generalizable to other populations. Third, since the GWAS meta-analysis of exposures did not include sex-specific genetic data, only genetic data for OA in each sex were used to conduct sex-specific analysis in our MR analyses. The sex-specific patterns of association between exposures and OA provide interesting clues and may provoke more subsequent investigations. Furthermore, GWAS meta-analysis is warranted to conduct sex-stratified analyses, especially when sex plays a role in the disorder.

## 5. Conclusions

This MR study provided evidence that genetically predicted homocysteine was causally associated with an increased risk of OA, especially at weight-bearing joints and in females. Folate and vitamin B12 had suggestively protective effects on OA. Homocysteine-lowering interventions, for example, through folate and vitamin B12 supplementation, may be a potential option for the treatment and prevention of OA, especially OA at weight-bearing joints and in females.

## Figures and Tables

**Table 1:** Characteristics of SNPs for homocysteine, folate, and vitamin B12.

Trait	SNP	Chromosome:Position	Gene	EA	OA	EAF	Beta	SE	*p*
Homocysteine	rs957140	11:89201627	NOX4	A	G	0.45	−0.045	0.008	2.43 × 10^−10^
	rs9369898	6:49382193	MUT	A	G	0.62	0.0449	0.007	2.17 × 10^−10^
	rs7130284	11:89148372	NOX4	T	C	0.07	−0.1242	0.013	1.88 × 10^−20^
	rs4660306	1:45978675	MMACHC	T	C	0.33	0.0435	0.007	2.33 × 10^−9^
	rs42648	7:89977760	GTPBP10	A	G	0.40	−0.0395	0.007	1.97 × 10^−8^
	rs2851391	21:44487404	CBS	T	C	0.47	0.056	0.008	1.70 × 10^−12^
	rs234709	21:44486964	CBS	T	C	0.45	−0.0718	0.007	3.90 × 10^−24^
	rs2275565	1:237048676	MTR	T	G	0.21	−0.0542	0.009	1.96 × 10^−10^
	rs1801222	10:17156151	CUBN	A	G	0.34	0.0453	0.007	8.43 × 10^−10^
	rs12780845	10:17223244	CUBN	A	G	0.65	0.0529	0.009	7.80 × 10^−10^
	rs12134663	1:11838646	MTHFR	A	C	0.80	−0.101	0.011	2.54 × 10^−21^
Folate	rs17421511	1:11857788	MTHFR	G	A	0.827	0.098	0.012	1.80 × 10^−15^
	rs652197	11:71849741	FOLR3	C	T	0.179	0.069	0.011	2.50 × 10^−10^
Vitamin B12	rs5753231	22:31003069	TCN2	C	T	0.790	0.064	0.010	7.50 × 10^−10^
	rs56077122	10:17207015	TRDMT1	A	C	0.335	0.087	0.009	4.80 × 10^−21^
	rs41281112	13:100518634	CLYBL	C	T	0.948	0.17	0.016	9.60 × 10^−27^
	rs34528912	11:59631535	TCN1	T	C	0.036	0.17	0.021	2.10 × 10^−15^
	rs2336573	19:8367709	CD320	T	C	0.031	0.32	0.021	1.10 × 10^−51^
	rs1801222	10:17156151	CUBN	G	A	0.593	0.11	0.007	1.10 × 10^−52^
	rs117456053	11:59616831	TCN1	G	A	0.976	0.16	0.027	1.90 × 10^−9^
	rs1141321	6:49412433	MUT	C	T	0.627	0.061	0.007	1.40 × 10^−16^

Gene = nearest gene to the SNP; EA = effect allele; EAF, effect allele frequency; beta = per allele effect on exposures; SE = standard error; *p* = *p* value for the genetic association.

## Data Availability

The GWAS summary data of OA was downloaded from the ‘Downloads’ page of the Musculoskeletal Knowledge Portal (https://mskkp.org, accessed on 4 December 2022), provided by Boer’s group. The GWAS summary data of folate and vitamin B12 were downloaded from GWAS Catalog website (https://www.ebi.ac.uk/gwas/, accessed on 4 December 2022).

## Supplementary Materials

The following supporting information can be downloaded at: https://www.mdpi.com/article/10.3390/nu15071636/s1, Figure S1: Scatter plot of the MR estimates for the association of homocysteine with the risk of OA and subtypes. (a) Overall OA; (b) knee OA; (c) hip OA; (d) spine OA; (e) hand OA; (f) thumb OA. The x-axis represents the effect size of SNPs on homocysteine; the y-axis represents the effect size of SNPs on OA and subtypes. Colors of the fitted line represent the four approaches used in univariable MR analyses. Figure S2: Leave-one-out analysis for MR analysis of the causal effect of homocysteine on OA and subtypes. (a) Overall OA; (b) knee OA; (c) hip OA; (d) spine OA; (e) hand OA; (f) thumb OA. Figure S3: Scatter plot of the MR estimates for the association of vitamin B12 with the risk of OA and subtypes. (a) Overall OA; (b) knee OA; (c) hip OA; (d) spine OA; (e) hand OA; (f) thumb OA. The x-axis represents the effect size of SNPs on vitamin B12; the y-axis represents the effect size of SNPs on OA and subtypes. Colors of the fitted line represent the four approaches used in univariable MR analyses. Figure S4: Leave-one-out analysis for MR analysis of the causal effect of vitamin B12 on OA and subtypes. (a) Overall OA; (b) knee OA; (c) hip OA; (d) spine OA; (e) hand OA; (f) thumb OA. Figure S5: Causal effect of homocysteine in OA in females in fixed-effects and random-effects IVW analyses. OR: odds ratio; CI: confidence interval; *p* value: *p* value of the causal estimate. Figure S6: Causal effect of folate in OA in females in fixed-effects and random-effects IVW analyses. OR: odds ratio; CI: confidence interval; *p* value: *p* value of the causal estimate. Figure S7: Causal effect of vitamin B12 in OA in females in fixed-effects and random-effects IVW analyses. OR: odds ratio; CI: confidence interval; *p* value: *p* value of the causal estimate. Figure S8: Causal effect of homocysteine in OA in males in fixed-effects and random-effects IVW analyses. OR: odds ratio; CI: confidence interval; *p* value: *p* value of the causal estimate. Figure S9: Causal effect of folate in OA in males in fixed-effects and random-effects IVW analyses. OR: odds ratio; CI: confidence interval; *p* value: *p* value of the causal estimate. Figure S10: Causal effect of vitamin B12 in OA in males in fixed-effects and random-effects IVW analyses. OR: odds ratio; CI: confidence interval; *p* value: *p* value of the causal estimate. Table S1: Detailed information on the genome-wide association study of OA. Table S2: Association (*p* < 5 × 10^−8^) of SNPs used as candidate genetic instruments for OA at any site with confounders or OA. Table S3: Results of overall MR Steiger direction test. Table S4: The summary information for instrumental variables of outcomes. Table S5: Cochran’s Q test for heterogeneity (MR-IVW). Table S6: Assessing directional pleiotropy through MR-Egger intercept and MR-PRESSO test. Table S7: Weighted median and MR-Egger methods for genetic associations between exposures and outcomes.
