# Supplementary material for: Associations of Homocysteine, Folate, and Vitamin B12 with Osteoarthritis: A Mendelian Randomization Study
Source: Nutrients. 2023 Mar 28;15(7):1636. doi: 10.3390/nu15071636 (PMC10096814; doi:10.3390/nu15071636)
Supplement: Supplementary file 1 [file nutrients-15-01636-s001.zip › Supplementary figures.pdf]

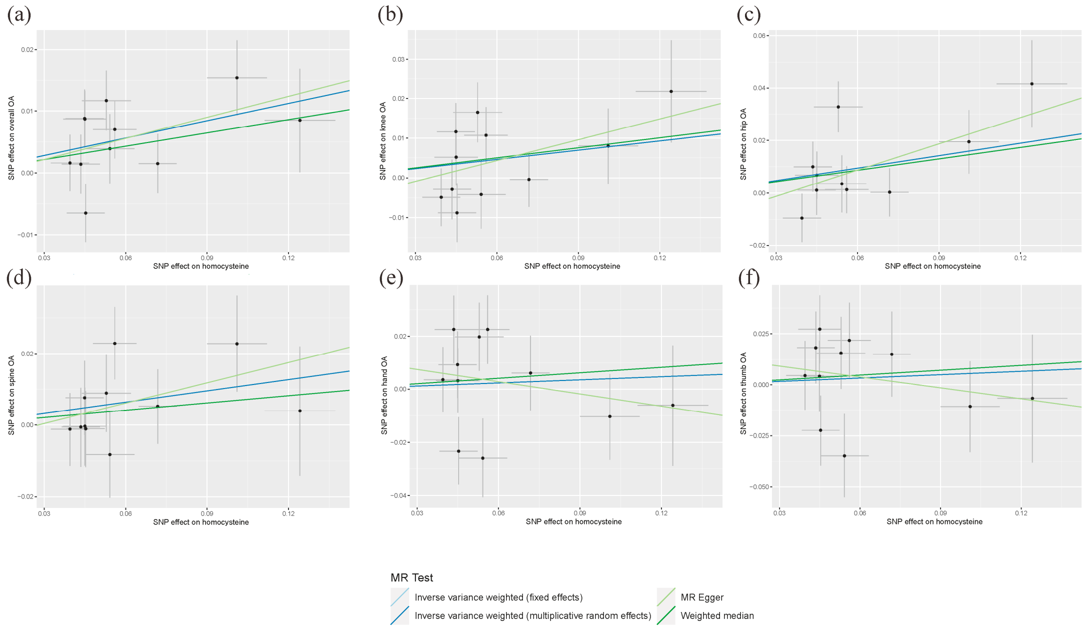

Figure S1: Scatter plot of the MR estimates for the association of homocysteine with risk of OA and subtypes. (a) overall OA; (b) knee OA; (c) hip OA; (d) spine OA; (e) hand OA; (f) thumb OA. The x-axis represents the effect size of SNPs on homocysteine; the y-axis represents the effect size of SNPs on OA and subtypes. Colors of fitted line represents for four approaches used in univariable MR analyses.

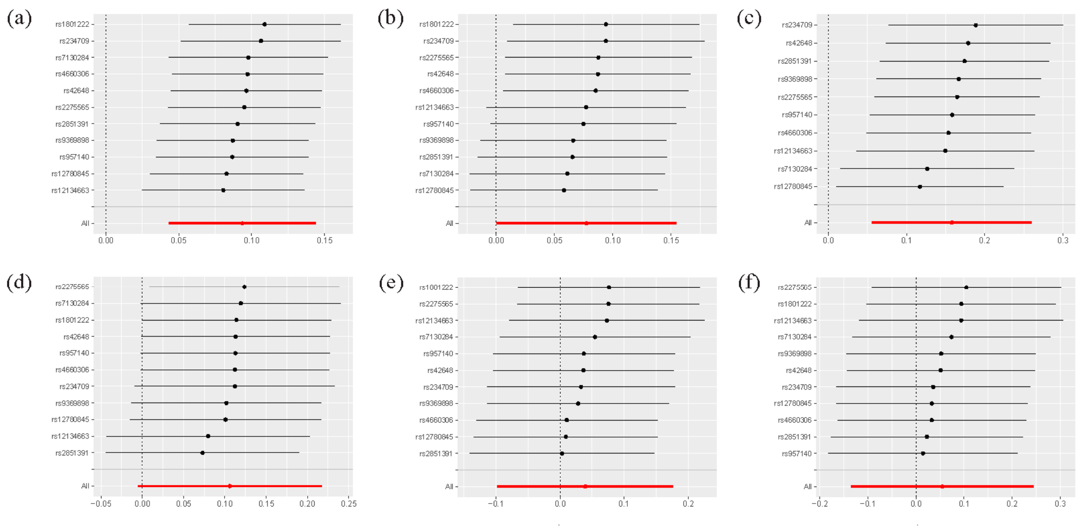

Figure S2: Leave-one-out analysis for MR analysis of the causal effect of homocysteine on OA and subtypes. (a) overall OA; (b) knee OA; (c) hip OA; (d) spine OA; (e) hand OA; (f) thumb OA.

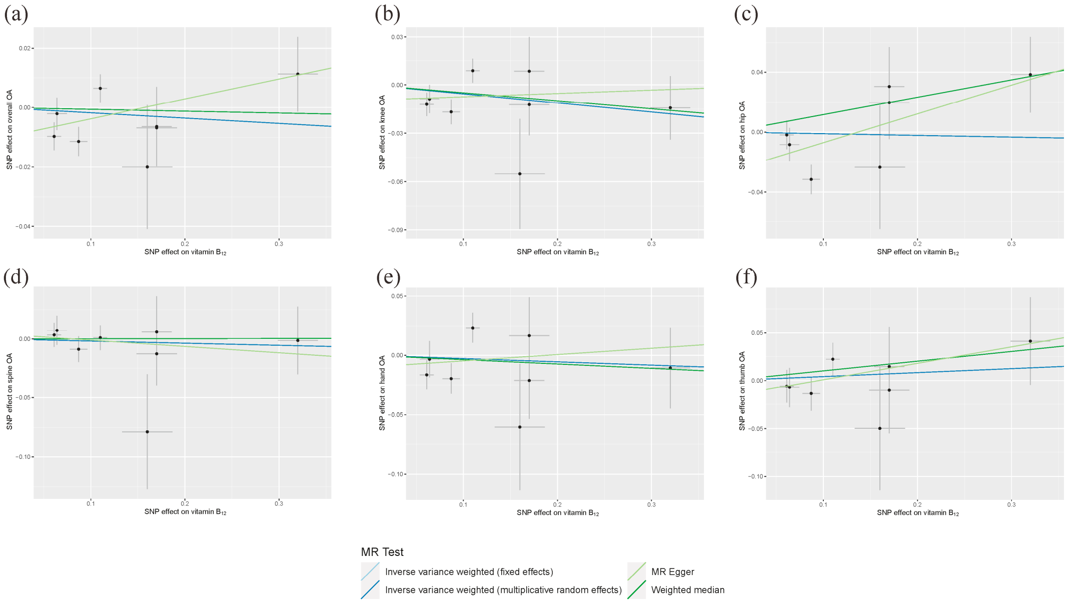

Figure S3: Scatter plot of the MR estimates for the association of vitamin B_{12} with risk of OA and subtypes. (a) overall OA; (b) knee OA; (c) hip OA; (d) spine OA; (e) hand OA; (f) thumb OA. The x-axis represents the effect size of SNPs on vitamin B_{12}; the y-axis represents the effect size of SNPs on OA and subtypes. Colors of fitted line represents for four approaches used in univariable MR analyses.

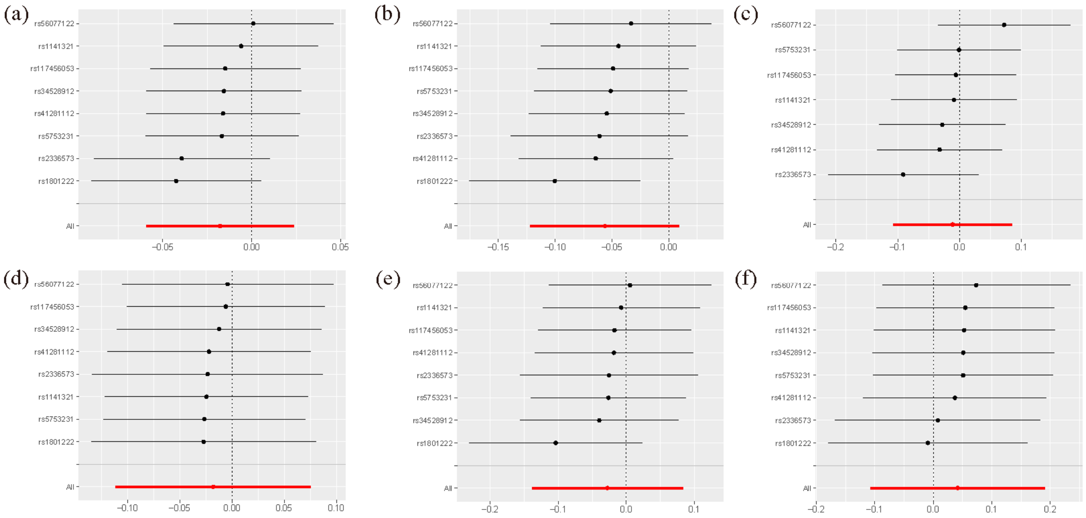

Figure S4: Leave-one-out analysis for MR analysis of the causal effect of Vitamin B_{12} on OA and subtypes. (a) overall OA; (b) knee OA; (c) hip OA; (d) spine OA; (e) hand OA; (f) thumb OA.

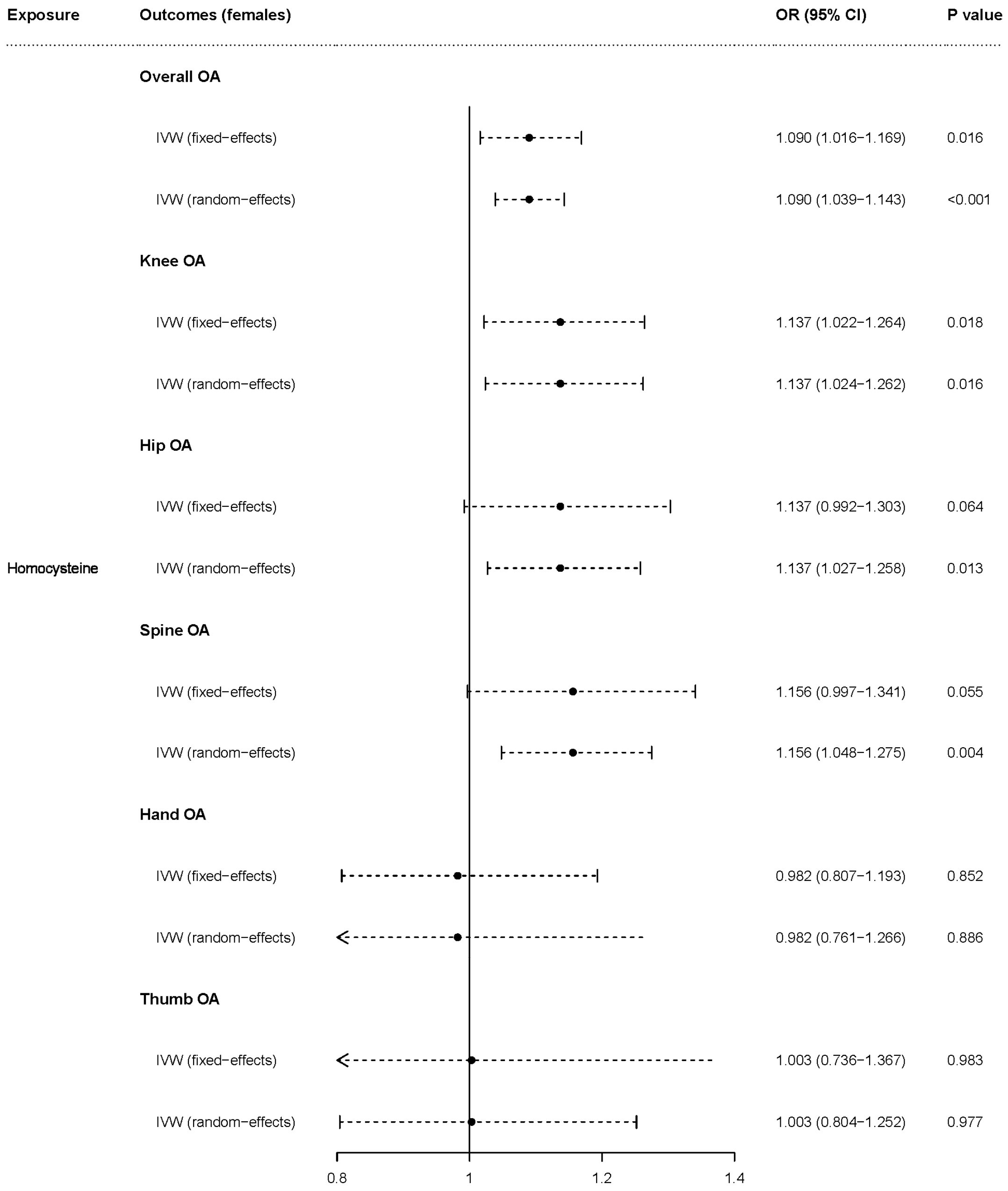

Figure S5: Causal effect of homocysteine on OA in females in fixed-effects and random-effects IVW analysis. OR: odds ratio; CI: confidence interval; P value: P value of the causal estimate.

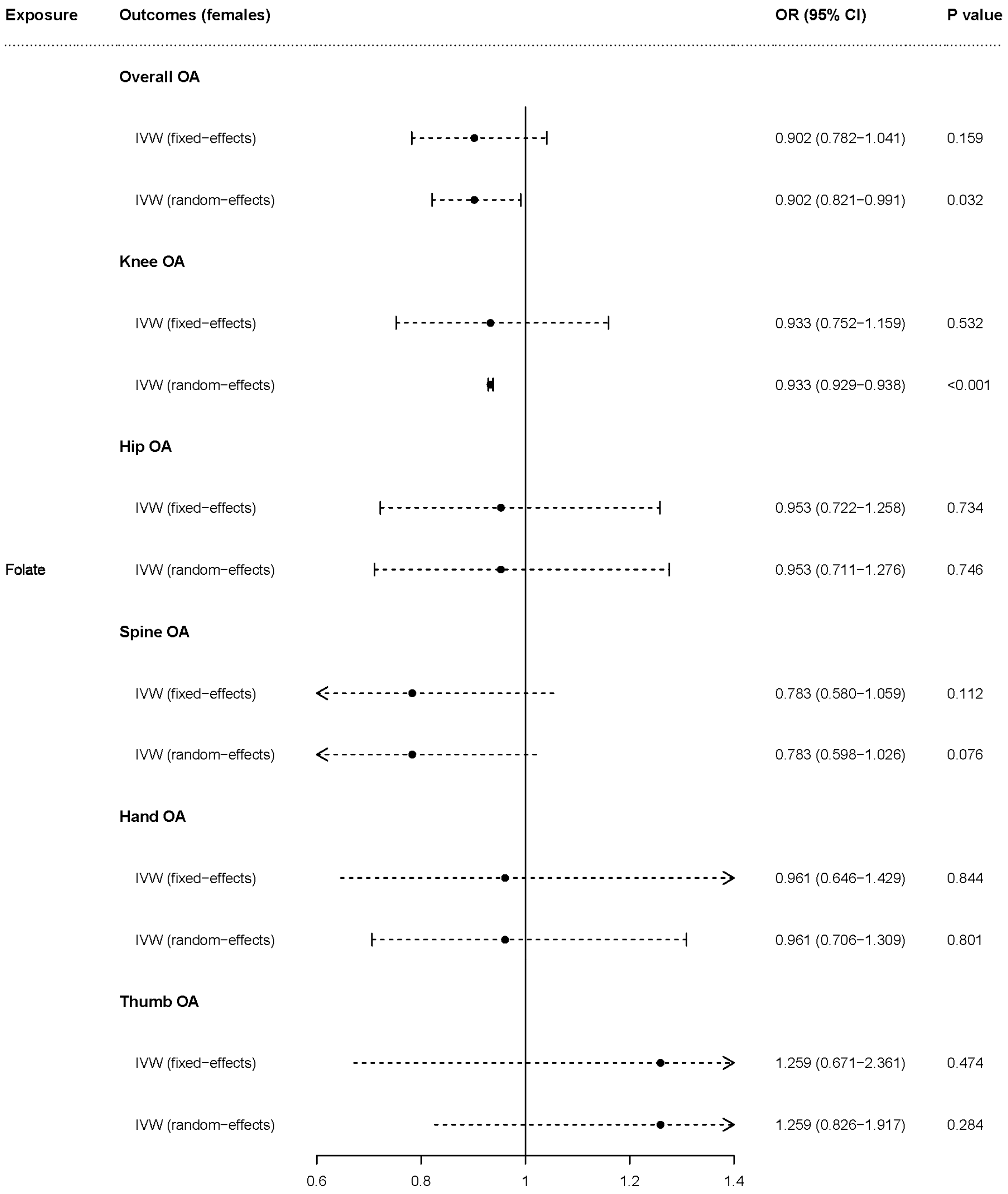

Figure S6: Causal effect of folate on OA in females in fixed-effects and random-effects IVW analysis. OR: odds ratio; CI: confidence interval; P value: P value of the causal estimate.

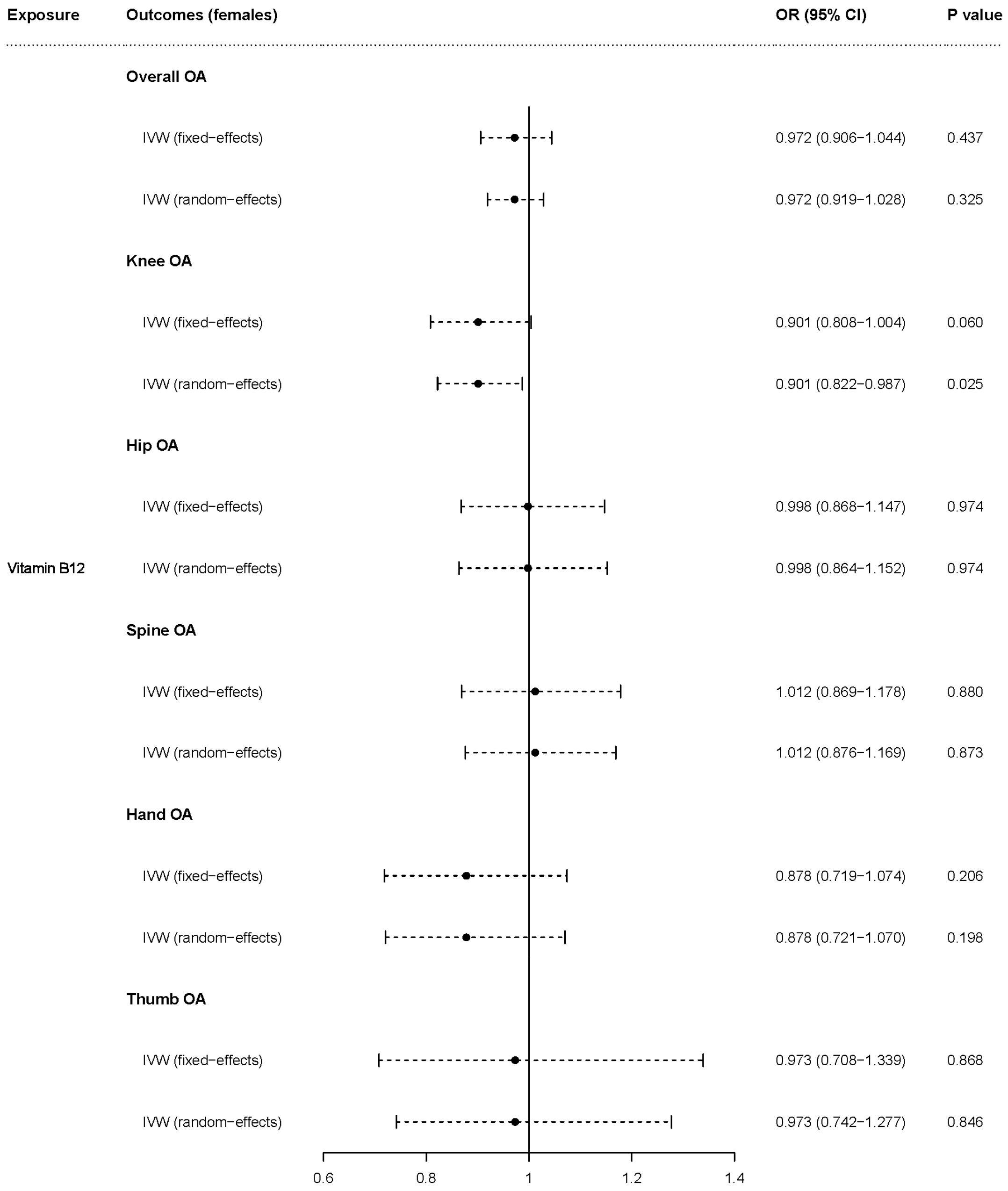

Figure S7: Causal effect of vitamin B_{12} on OA in females in fixed-effects and random-effects IVW analysis. OR: odds ratio; CI: confidence interval; P value: P value of the causal estimate.

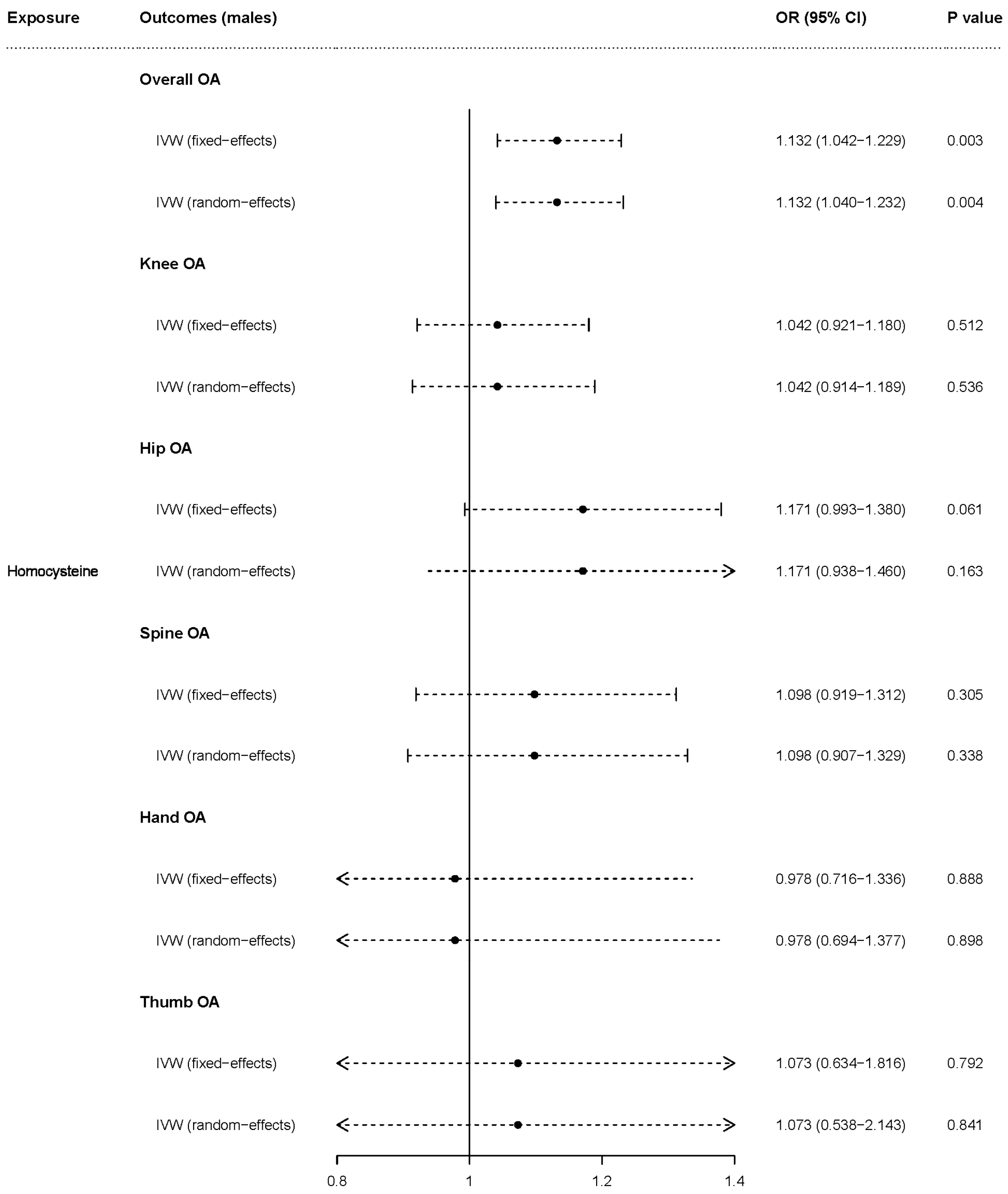

Figure S8: Causal effect of homocysteine on OA in males in fixed-effects and random-effects IVW analysis. OR: odds ratio; CI: confidence interval; P value: P value of the causal estimate.

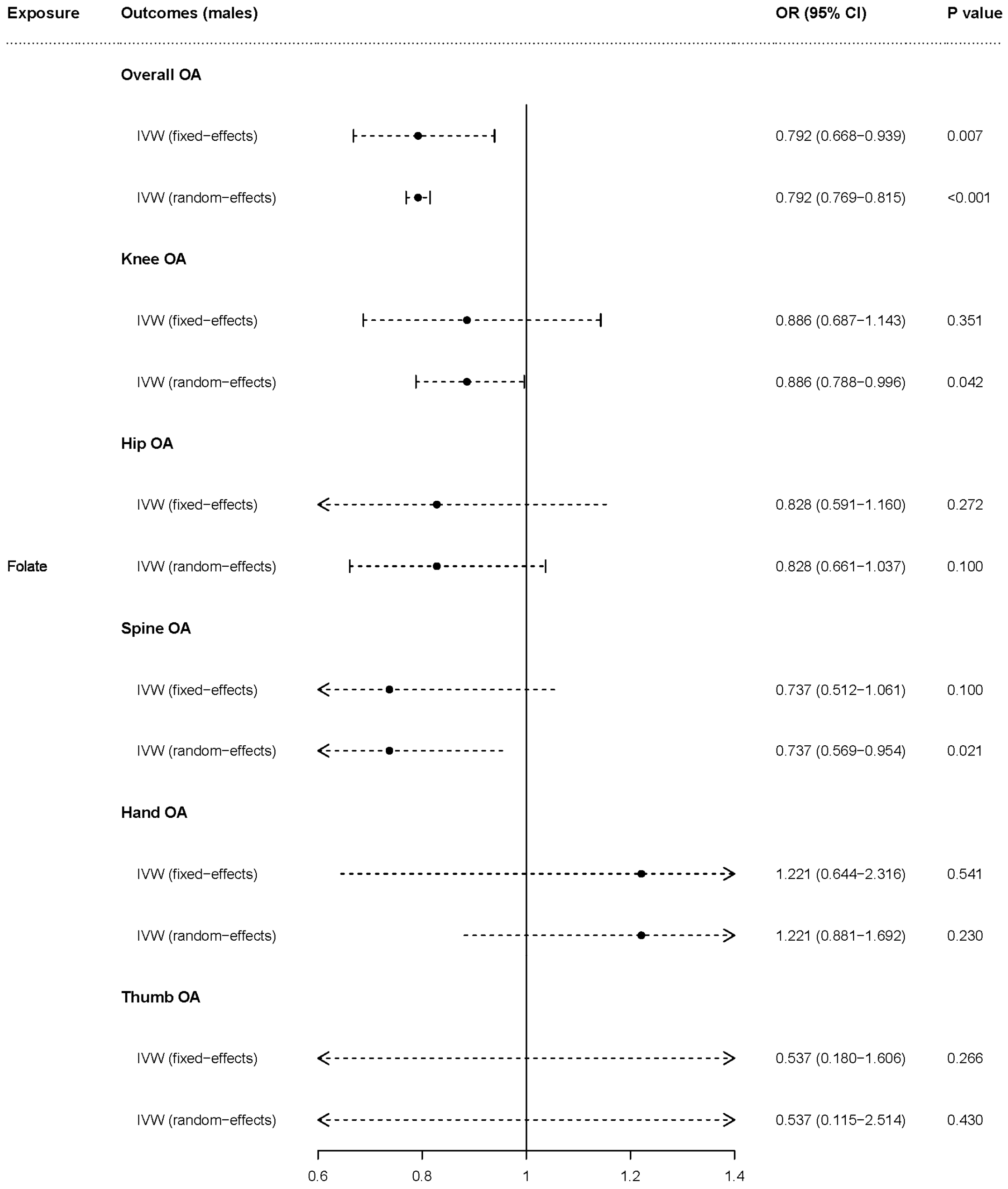

Figure S9: Causal effect of folate on OA in males in fixed-effects and random-effects IVW analysis. OR: odds ratio; CI: confidence interval; P value: P value of the causal estimate.

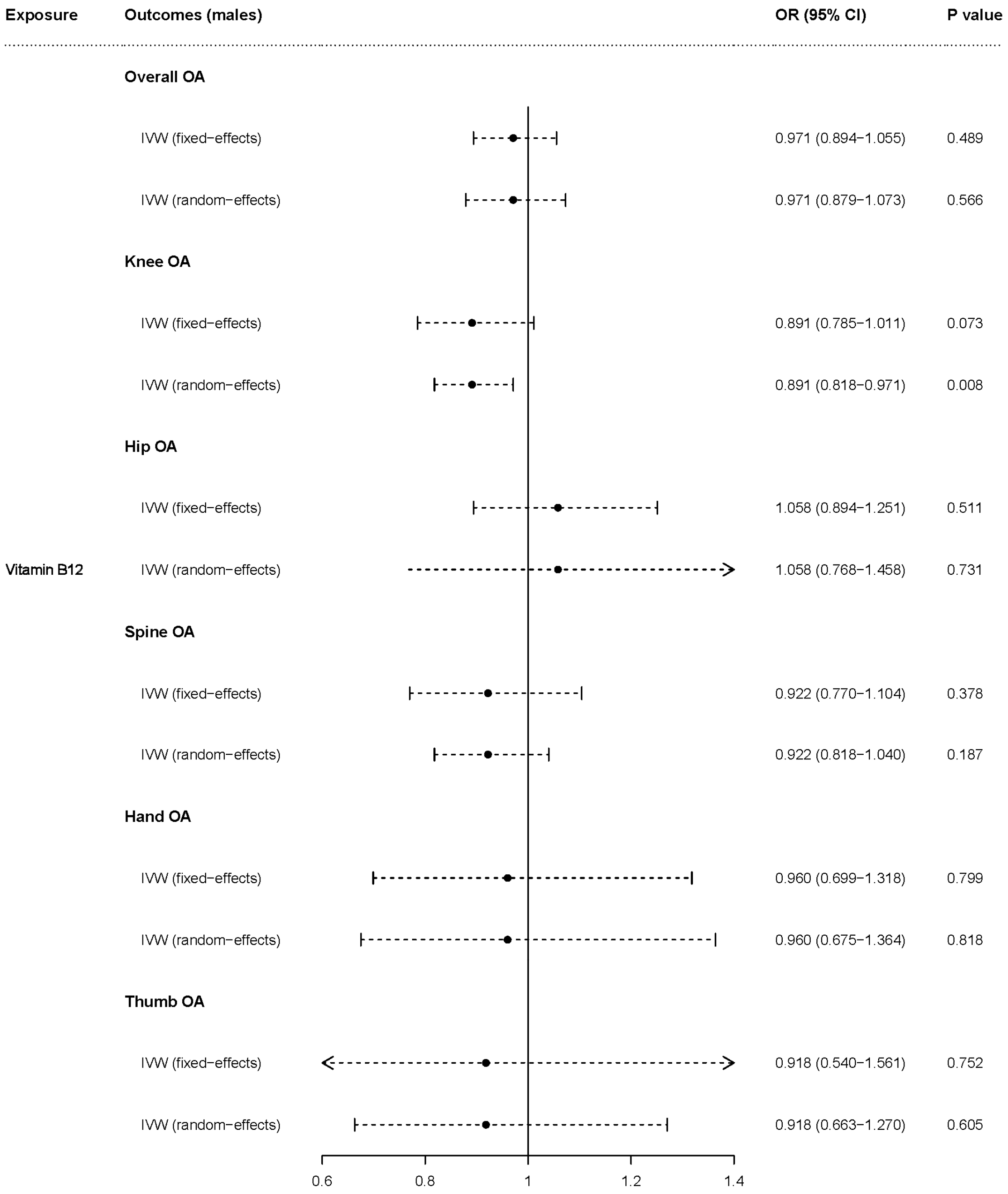

Figure S10: Causal effect of vitamin B12 on OA in males in fixed-effects and random-effects IVW analysis. OR: odds ratio; CI: confidence interval; P value: P value of the causal estimate.
